# The Effect of Intraoperative Restricted Normal Saline during Orthotopic Liver Transplantation on Amount of Administered Sodium Bicarbonate

**Published:** 2014-05

**Authors:** Mohammad Ali Sahmeddini, Farahzad Janatmakan, Mohammad Bagher Khosravi, Sina Ghaffaripour, Mohammad Hossein Eghbal, Sakine Shokrizadeh

**Affiliations:** 1 Shiraz Anesthesiology and Critical Care Research Center, Nemazee Hospital, Shiraz University of Medical Sciences, Shiraz, Iran;; 2 Department of Anesthesiology and Critical Care, Ahvaz Jundishapur University of Medical Sciences, Ahvaz, Iran

**Keywords:** Acidosis, Liver transplantation, Sodium bicarbonate, Crystalloid solution

## Abstract

**Background: **Severe metabolic acidosis occurs during orthotopic liver transplantation (OLT) particularly during the anhepatic phase. Although NaHCO_3_ is considered as the current standard therapy, there are numerous adverse effects. The aim of this study was to determine whether the restricted use of normal saline during anesthesia could reduce the need for NaHCO_3_.

**Methods: **In this study we enrolled 75 patients with end-stage liver disease who underwent OLT from February 2010 until September 2010 at the Shiraz Organ Transplantation Center. Fluid management of two different transplant anesthetics were compared. The effect of restricted normal saline fluid was compared with non-restricted normal saline fluid on hemodynamic and acid-base parameters at three times during OLT: after the skin incision (T1), 15 min before reperfusion (T2), and 5 min after reperfusion (T3).

**Results: **There were no significant differences in demographic characteristics of the donors and recipients (P>0.05). In the restricted normal saline group there was significantly lower central venous pressure (CVP) than in the non-restricted normal saline group (P=0.002). No significant differences were noted in the other hemodynamic parameters between the two groups (P>0.05). In the non-restricted normal saline group arterial blood pH (P=0.01) and HCO_3_ (P=0.0001) were significantly less than the restricted normal saline group. The NaHCO_3_ requirement before reperfusion was significantly more than with the restricted normal saline group (P=0.001).

**Conclusion: **Restricted normal saline administration during OLT reduced the severity of metabolic acidosis and the need for NaHCO_3_ during the anhepatic phase.

**Trial Registration Number:** IRCT2013110711662N5

## Introduction

The crucial issue during liver transplantation surgery is progressive metabolic acidosis. This form of acidosis begins during the dissection phase and increases during the anhepatic phase.^1^^,^^2^ In the dissection phase the major causes of this acidosis are crystalloid therapy and hypotension, the latter results from drainage of ascites fluid, dissection and mobilization of the liver.^1^^,^^3^ In the anhepatic phase, the major cause of acidosis is lactic acidosis due to the accumulation of lactic acid.^2^ Metabolic acidosis begins to subside several minutes after reperfusion of the new liver, which is a sign of graft function.^4^

One of the important factors attributed to metabolic acidosis during anesthesia for liver transplantation is administration of large quantities of sodium chloride-containing fluids for maintenance of the hemodynamic state. This type of fluid decreases the difference between the total concentrations of strong cations and anions [the strong ion difference or (SID)] which causes metabolic acidosis.^5^^-^^7^ Currently NaHCO_3_ is the standard treatment for metabolic acidosis during orthotopic liver transplantation (OLT).^8^

With the use of NaHCO_3_ there are complications such as increases in serum sodium and serum osmolarity, exacerbation of intracellular acidosis and increases in plasma lactate.^9^^,^^10^ It seems straightforward that administration of NaHCO_3_ to acidic blood will easily raise the pH, however in reality, it is more sophisticated.^11^^,^^12^

The aim of the present study was to compare the effect of restricted crystalloid therapy with non-restricted crystalloid therapy during anesthesia for OLT on the severity of metabolic acidosis and the amount of NaHCO_3_ usage at the end of the anhepatic phase.

## Patients and Methods

In this randomized controlled trial (IRCT ID: IRCT2013110711662N5) we enrolled 75 patients with end-stage liver disease who underwent orthotropic deceased donor liver transplantations from February 2010 to September 2010 in the Shiraz Organ Transplantation Center. We compared fluid managements of two different transplant anesthetics between the two groups: restricted normal saline and non-restricted normal saline. After receiving approval from the Institutional Ethics Committee, written informed consent was obtained from the patients. The patients were randomly allocated into two groups according to the anesthesiologists’ work shifts.

Eligible patients included all adult patients with end-stage liver disease above the age of 16 years who were selected for OLT. Patients with hepatorenal syndrome types І and ІІ, baseline serum potassium >6 meq/l, hepatopulmonary syndrome, portopulmonary hypertension, ejection fraction <60% and cirrhotic cardiomyopathy were excluded from study.

The induction of anesthesia was done with thiopental (3-5 mg/kg), fentanyl (2 μg/kg), and midazolam (0.03 mg/kg); pancuronium (0.1 mg/kg) was used for neuromuscular blockade. Anesthesia was maintained with isoflurane plus a 50% air-50% oxygen mixture and ventilation adjusted to maintain an end-tidal CO_2_ of 30-35 mmHg. Cardiovascular function was monitored using an electrocardiogram, a radial artery catheter, and the central venous pressure (CVP) through the right internal jugular vein with a double lumen spectrophotometer catheter no. 12.

Patients in the restricted normal saline group received 5 ml/kg/h normal saline as maintenance fluid therapy. Moreover, patients received 5% albumin, fresh frozen plasma and packed cells to maintain CVP at ≥80% of baseline values and the hematocrit levels at approximately 30% during anesthesia for the hepatectomy and anhepatic phase. If drainage of ascitic fluid in patients with ascites caused hypotension, we began a norepinephrine infusion to correct for hypotension. The ascites fluid was not replaced with normal saline. At the start of the portal vein anastomosis, arterial blood gas levels were checked in all patients. If the base excess (BE) was ≤-3, sodium bicarbonate was used to correct for metabolic acidosis.

In the non-restricted normal saline group, patients received 10 ml/kg/h normal saline as the maintenance fluid therapy. In addition, 25% albumin and packed cells were given to maintain the CVP at ≥80% baseline values and hematocrit levels at approximately 30% during anesthesia for hepatectomy and the anhepatic phase. In case of draining ascites fluid, this fluid was replaced with normal saline and 25% albumin to maintain blood pressure. At the start of portal vein anastomosis, for all patients, we checked arterial blood gas levels. In cases with BE ≤-3, sodium bicarbonate was given to correct for metabolic acidosis.

In both groups, in cases of decreased mean blood pressure (MAP) to <60 mmHg despite adequate fluid therapy, we administered norepinephrine at an initial dose of 0.05 μ/kg/min; the dosage was increased until MAP was maintained at levels above 60 mmHg. 

The primary outcome was sodium bicarbonate dosage used to correct metabolic acidosis at the end of the anhepatic phase. Secondary outcomes were hemodynamic (MAP and heart rate per min) after declamping the portal vein and urine output at the end of the hepatectomy, anhepatic and neo-hepatic phases. 

Laboratory data collected during the procedure included arterial blood gas values of arterial pH, partial oxygen pressure (PaO_2_), partial carbon dioxide pressure (PaCO_2_), standard bicarbonate (HCO_3_) and BE values. We performed the measurements at three times: after the skin incision (baseline, T1); 15 min before reperfusion (T2), and 5 min after reperfusion (T3).

Treatment with blood and blood components for both groups were recorded and compared with each other.

We defined the total ischemic time as the period from the aortic cross-clamping and perfusion with the preservation solutions in the donor to the completion of the anastomosis with portal reperfusion.

*Statistical Analysis*

By using the power static software collection (SSC), with a power of 80%, an α level of 0.05, consideration of variance 15 and mean difference 10 in BE, the appropriate sample size for each group was determined to be at least 34 patients (total of 68 patients).

Analysis was performed using SPSS 14.0 version for Mac OS X. Discrete variables were compared using the chi-square and Fisher’s exact tests using a permutation method for multiple testing. Continuous variables were compared by the independent t-test and repeated measures analysis of variance. All values have been presented as mean±SD and P<0.05 was considered significant.

## Results

**Figure 1:**
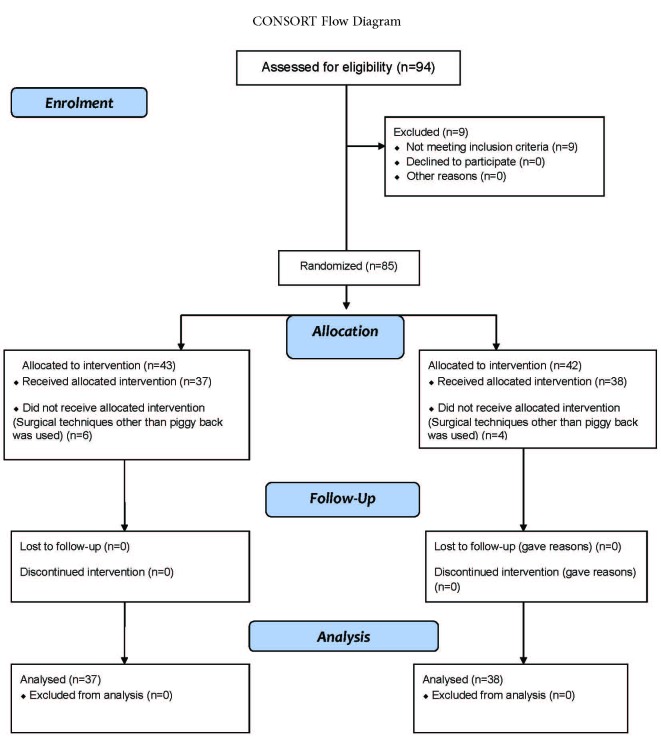
Flowchart of patients according to the consort guideline

From 94 patients with end-stage liver disease who underwent orthotropic deceased donor liver transplantation from February 2010 to September 2010, 75 were eligible for this study. There were 37 patients assigned to the restricted normal saline group and 38 in the non-restricted normal saline group (figure 1).

**Figure 2:**
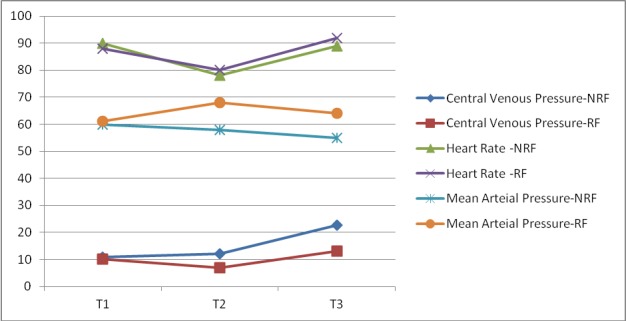
Mean changes in heart rate, central venous pressure (CVP) and mean arterial pressure from baseline to reperfusion in the non-restricted normal saline (NRF) group and restricted normal saline (RF) group. T1: After skin incision; T2: 15 min before reperfusion; T3: 5 min after reperfusion.

There were no significant differences in the demographic characteristics of the donors and the recipients (P>0.05; tables 1 and 2) or in the graft factors between both groups (P>0.05; table 2). We observed no significant differences in the baseline mean arterial pressure (P=0.22), the baseline heart rate (P=0.71) and the baseline CVP (P=0.32) between the two groups (figure 2).

**Table 1:** Demographics characteristics of participants

	**Crystalloid restricted (n=37)**	**Crystalloid non-restricted (n=38)**	**P value**
Sex(M/F)	20/17	22/16	0.09
Age (years)	27±5	26±8	0.72
MELD score	20±3	19±2	0.59
Body weight (kg)	65±7	59±9	0.08
Height (cm)	162±9	169±7	0.83

All values reported represent the mean±standard deviation.

**Table 2:** Demographic characteristics of donor groups

	**Crystalloid restricted (n=37)**	**Crystalloid non-restricted (n=38)**	**P value**
Donor age (years)	28±7	30±6	0.31
Fatty change in liver (%)	4.50±1.8	5.10±0.6	0.66
Graft weigh/recipient weight	1.15±0.18	1.20±0.25	0.45
Total ischemic time (h)	9.5±1.8	10.8±1.4	0.28

All values reported represent the mean**±**standard deviation.

There were no significant differences in estimated blood loss and use of blood products during surgery between the two groups (P>0.05; table 3).

**Table 3:** Blood and blood component therapy in both groups

**Variables**	**Crystalloid fluid restricted (n=37)**	**Crystalloid fluid non-restricted (n=38)**	**P value**
Estimated blood loss (ml)	2100 (1500-5500)	2500 (1600-6000)	0.34
PRBC (ml)	2000 (1250-4500)	2250 (1500-5000)	0.11
FFP (ml)	1000 (500-15000)	250 (0-1250)	0.09
Total volume of normal saline	3000 (2500-3250)	6000 (5000-6500)	0.001

During the hepatectomy, anhepatic and neo-hepatic phases, the restricted normal saline group had lower CVP compared to the non-restricted normal saline group (P=0.002; figure 2). However, no significant differences were found between the two groups for urine output (ml/kg/h) during the three phases of OLT (P>0.05; table 4). In addition, during these phases there were no significant differences in mean arterial pressures and the heart rates between the two groups (P=0.34, P=0.47 respectively; figure 2).

**Table 4:** Urine output during the three stages of liver transplantation

**Variables**	**Crystalloid fluid restricted (n=37)**	**Crystalloid fluid ** **non-restricted (n=38)**	**P value**	**95% CI **
Urine output in hepatectomy phase (ml/kg/h)	0.53±0.15	0.49±0.31	0.65	-0.11 to 0.31
Urine output in anhepatic phase (ml/kg/h)	0.25±0.28	0.31±0.11	0.45	-0.15 to 0.18
Urine output in neo-hepatic phase (ml/kg/h)	0.81±0.67	1.01±0.21	0.58	-0.5 to 0.41

All values are reported as mean±standard deviation. CI: Confidence interval

**Figure 3:**
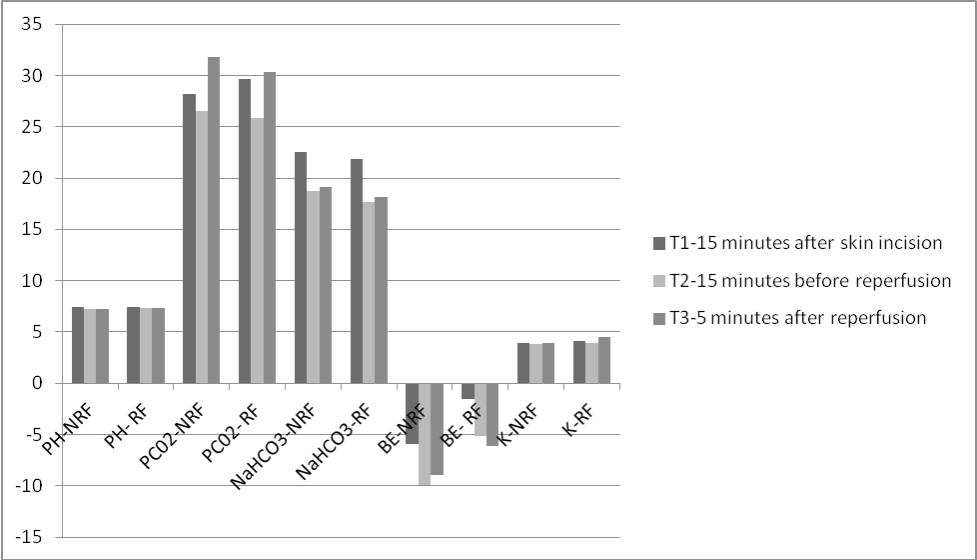
Mean changes in pH, PCO2, NaHCO3 and serum potassium (K) from baseline to reperfusion in the non-restricted normal saline (NRF) and restricted normal saline (RF) groups.

We observed no significant differences in baseline arterial blood pH (P=0.32), HCO_3_ (P=0.47), and BE (P=0.67; figure 3). However, at the end of the hepatectomy and anhepatic phases in the restricted normal saline group compared to the non-restricted normal saline group, the changes in arterial blood pH (P=0.01), HCO_3_ (P=0.001), and BE (P=0.02; figure 3) were less marked. The NaHCO_3_ requirement before reperfusion in the non-restricted normal saline group was 200.44±18.21 whereas in the restricted normal saline group this requirement before reperfusion was 0±0.0 (P=0.001). We observed no significant differences in arterial blood pH (P=0.78), HCO_3_ (P=0.12), and BE (P=0.59) after reperfusion between the two groups (figure 3).

## Discussion

The present study showed that smaller volumes of normal saline fluid used during OLT anesthesia led to decreased severity of metabolic acidosis and a decrease in the cumulative dose of NaHCO_3_.

During liver transplant surgery one major problem is progressive metabolic acidosis, which starts during the dissection stage and accelerates during the anhepatic phase.^1^ As Ali et al. have shown in their study, due to the complex pathophysiology of end-stage liver disease it is better to consider the effect of the difference between the total concentrations of strong cations and anions (SID), the total concentration of weak acids, and the PaCO_2_ amounts on blood pH for diagnosis and management of the acid-base changes during liver transplantation.^2^ Thus, the current study has confirmed that restricted normal saline fluid use could decrease SID and prevent progressive metabolic acidosis during the hepatectomy and anhepatic phases of OLT.

It must be considered that during general anesthesia (GA), low SVR of end-stage liver disease aggravated by the inherent vasodilating property of anesthetic agents.^13^ The resultant profound decrease in SVR, drainage of ascitic fluid and bleeding during hepatectomy will lead to significant hypotension that requires substantial volumes of fluid administration and vasoconstrictor drugs.^14^^,^^15^ Based on a study by Schroeder et al., administration of large quantities of sodium chloride-containing fluids for maintenance of the hemodynamic decrease the SID, which in turn leads to a lower pH.^16^ However in our study we have used more colloid fluid instead of a substantial volume of crystalloid fluid in the restricted normal saline fluid group. Therefore at the end of the anhepatic phase, sodium bicarbonate demand was decreased.

According to previous studies administration of large quantities of normal saline fluid during OLT can lead to progressive metabolic acidosis.^13^^,^^17^ Therefore sodium bicarbonate use at end of the anhepatic phase is inevitable.^16^ However, complications exist with the administration of exogenous sodium bicarbonate for correction of metabolic acidosis. Administration of sodium bicarbonate increases the SID which tends to raise the pH because sodium is a strong cation and bicarbonate is not a strong ion. Simultaneously PaCO_2_ becomes elevated, which tends to cause a lower pH.^18^^,^^19^

In animal models of lactic acidosis, sodium bicarbonate did not predictably raise the arterial pH.^8^ In some studies, the pH remained constant or fell because bicarbonate infusion augmented the production of lactic acid.^18^ Mechanisms to explain this have included a shift in the oxyhemoglobin-saturation relationship and enhanced anaerobic glycolysis, which is perhaps mediated by the pH-sensitive, rate-limiting enzyme phosphofructokinase.^11^ Therefore, administration of sodium bicarbonate can not only correct lactic acidosis but also cause side effects such as fluid and sodium load.^20^ This can result in hypervolemia, hyperosmolarity, and hypernatremia. Hypernatremia is hazardous during liver transplantation, particularly in patients with low serum sodium levels who are at risk of central pontine myelinolysis due to rapid correction of hyponatremia.^21^

## Conclusion

The avoidance of large quantities of sodium chloride-containing fluids and use of colloid fluid to maintain a stable hemodynamic status decreases hyperchloremic acidosis during anesthesia for liver transplantation and reduces the need for sodium bicarbonate.
